# A Study on the Interactions of Proteinase K with Myricetin and Myricitrin by Multi-Spectroscopy and Molecular Modeling

**DOI:** 10.3390/ijms24065317

**Published:** 2023-03-10

**Authors:** Kefan Liu, Yubo Zhang, Wei Zhang, Liyan Liu, Zhan Yu

**Affiliations:** 1 College of Chemistry and Chemical Engineering, Shenyang Normal University, Shenyang 110034, China; 2 Provincial Key Laboratory for Separation and Analysis of Complex Systems in Liaoning Universities, Shenyang Normal University, Shenyang 110034, China

**Keywords:** proteinase K, myricetin, myricitrin, fluorescence quenching, molecular docking, molecular dynamics

## Abstract

Myricetin (MYR) and myricitrin (MYT) are well recognized for their nutraceutical value, such as antioxidant, hypoglycemic, and hypotensive effects. In this work, fluorescence spectroscopy and molecular modeling were adopted to investigate the conformational and stability changes of proteinase K (PK) in the presence of MYR and MYT. The experimental results showed that both MYR and MYT could quench fluorescence emission via a static quenching mechanism. Further investigation demonstrated that both hydrogen bonding and van der Waals forces play significant roles in the binding of complexes, which is consistent with the conclusions of molecular modeling. Synchronous fluorescence spectroscopy, Förster resonance energy transfer, and site-tagged competition experiments were performed to prove that the binding of MYR or MYT to PK could alter its micro-environment and conformation. Molecular docking results revealed that either MYR or MYT spontaneously interacted with PK at a single binding site via hydrogen bonding and hydrophobic interactions, which is consistent with the results of spectroscopic measurements. A 30 ns molecular dynamics simulation was conducted for both PK-MYR and PK-MYT complexes. The calculation results showed that no large structural distortions or interaction changes occurred during the entire simulation time span. The average RMSD changes of PK in PK-MYR and PK-MYT were 2.06 and 2.15 Å, respectively, indicating excellent stability of both complexes. The molecular simulation results suggested that both MYR and MYT could interact with PK spontaneously, which is in agreement with spectroscopic results. This agreement between experimental and theoretical results indicates that the method herein could be feasible and worthwhile for protein–ligand complex studies.

## 1. Introduction

**Figure 1:**
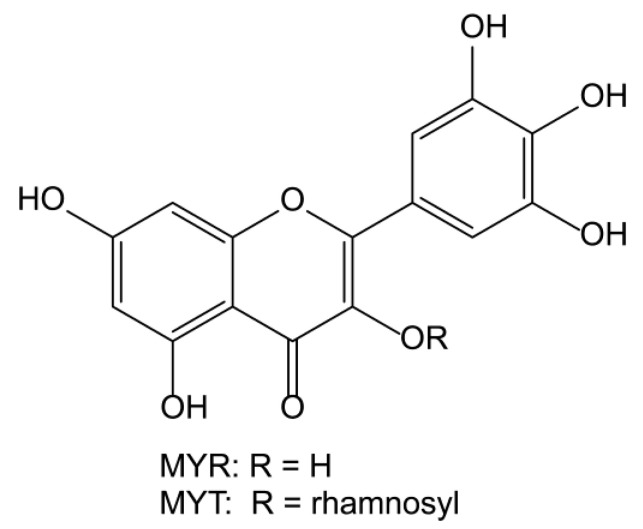
Chemical structures of MYR and MYT.

Myricetin (MYR, Figure 1) is a flavonoid compound found in the bark and leaves of Chinese bayberry (*Morella rubra*) family of plants [1], which has various pharmacological activities, such as acting as an antioxidant, protecting against cardiovascular disease, and having antitumor, antibacterial, hypoglycemic, lipid-regulating, and liver-protecting effects [2]. Bertin et al. [3] discovered that MYR has a strong antioxidant capacity and was able to effectively slow down the oxidation of free radicals in human low-density lipoprotein and vascular endothelial cells. Kim et al. [4] also found that MYR induced apoptosis in human colon cancer cells and may be useful in developing therapeutic agents for colon cancer. Myricitrin (MYT), a flavonol compound and a glycoside of MYR, is found in large quantities in fruits, bark, leaves, and other natural plants of the waxberry family [5]. It plays a key role in reducing blood sugar levels, blood pressure, and cytotoxicity. Zhang et al. [6] found that MYR significantly reduces blood glucose levels in alloxan-induced diabetic mice, and Yan et al. [7] demonstrated that MYT can inhibit IL-1β-induced extracellular matrix degradation in mouse chondrocytes, potentially making it useful in the treatment of osteoarthritis. Jo et al. showed that both MYR and MYT have prominent anti-African swine fever virus protease activity by forming protein–flavonoid complexes [8]. Vojta and coworkers demonstrated both MYR and MYT have intramolecular hydrogen bonds, which are important to their biological activities [9]. However, both MYR and MYT have low oral bioavailability due to their poor aqueous solubility, which limits their pharmaceutical use [10].

Proteinase K (PK) is a serine protease from the fungus *Tritirachium album* limber that belongs to the subtilisin family of enzymes [11]. It is a single-chain protein consisting of 279 amino acid residues and has a molecular weight of 28,930 Da with two disulfide bonds. PK is often used for spectroscopic assays due to its 2 tryptophan residues (Trp8 and Trp212) and 17 tyrosine residues, which are suitable for spectroscopic analysis [12]. It is commonly used to digest proteins, remove contaminants, and inactivate DNases and RNases in protein-free DNA or RNA samples.

Despite the widespread use of PK in various industries and scientific research, few studies have been conducted on the binding ability of flavonoids to PK. In order to better understand the potential molecular recognition between PK and typical flavonoid compounds, we used a combination of experimental and theoretical techniques including UV–visible absorption spectroscopy, fluorescence spectroscopy (FL), molecular docking, and molecular dynamic simulation methods to study the interactions between MYR/MYT and PK. The investigation of PK-MYR and PK-MYT binding mechanisms could provide valuable insights for the application of MYR and MYT to the food and pharmaceutical industries. Additionally, we hope that this study would provide valuable information to understand the impact of flavonoids on the structure and stability of PK.

## 2. Results and Discussion

### 2.1. Fluorescence Quenching Mechanism

Fluorescence spectroscopy is an effective method used to investigate conformational changes of proteins and binding parameters of ligands to fluorophore residues of proteins such as Trp, Tyr or Phe [13,14]. PK contains two Trp residues (Trp8 and Trp212), which make it possible to emit fluorescence. The fluorescence emission intensity of PK decreases gradually when MYR or MYT interacts with PK. The process by which this phenomenon occurs is called fluorescence quenching, which is the result of the altered microenvironment of PK. To eliminate the interference of fluorescence quenching from the inner filter effect, the experiments were corrected for fluorescence intensity using Equation (1).

**Figure 2:**
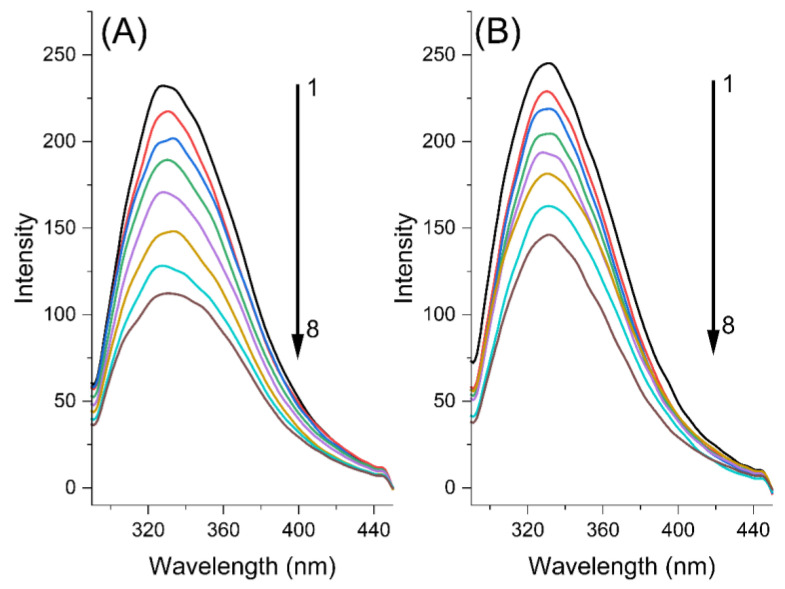
Fluorescence emission spectra of 3.46 μM PK at pH 8.0, in the presence of different concentrations of MYR (**A**) and MYT (**B**), respectively, at 300 K. From 1 to 8, the concentrations of MYR and MYT were 0.0, 2.5, 5.0, 7.0, 10.0, 15.0, 20.0, and 25.0 μM, respectively.

(1) Fcorr=Fobs×e(Aex+Aem)2

where *F_corr_* and *F_obs_* are the fluorescence emission intensity values of the corrected and pre-corrected systems, respectively. *A_ex_* and *A_em_* are the UV–Vis absorption intensities of quencher and PK at excitation and emission wavelengths, respectively. Figure 2 clearly shows that both MYR and MYT, in conjunction with PK, have a fluorescence emission peak at 330 nm under an excitation wavelength of 278 nm.

The fluorescence intensity of PK progressively decreased with the increase in temperature from 290 to 310 K. The higher the temperature, the lower the fluorescence intensity of PK. Meanwhile, a significant decrease in the fluorescence intensity of PK was investigated with increasing concentrations of MYR and MYT at a fixed concentration of PK, suggesting that PK strongly interacted with MYR and MYT, and the quencher resulted in a reduction of the hydrophobic microenvironment around the tryptophan residue of PK, producing PK–myricetin and PK–myricitrin complexes.

To investigate the differences in the fluorescence quenching patterns of the PK-MYR and PK-MYT complexes, the Stern–Volmer equation (Equation (2)) was used for analysis [15,16].

(2) $$\frac{F_{0}}{F}=1+K_{q}\tau_{0}\left[Q\right]=1+K_{\mathrm{sv}}[Q]$$

where *F* and *F*_0_ are the fluorescence emission intensities of PK in the presence or absence of the quencher (MYR or MYT), respectively, [*Q*] is the concentration of the quencher, *K*_sv_ is the ratio of the bimolecular quenching model constant to the single-molecule decay rate constant, namely, the Stern–Volmer equation quenching constants, *K*_q_ is the fluorescence quenching rate constant, and *τ*_0_ is the average lifetime of the fluorescent molecule when no quencher is added, while the average lifetime of biological macromolecules is 1 × 10^−^^8^ s [17].

**Figure 3:**
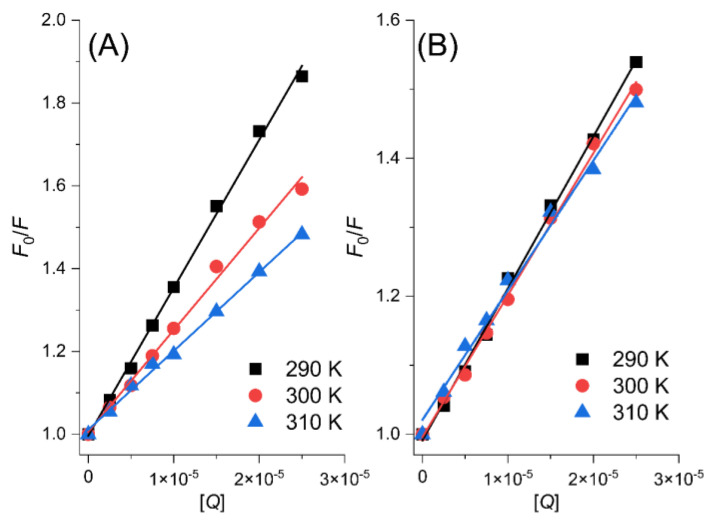
Stern-Volmer plots for PK-MYR (**A**) and PK-MYT (**B**).

Fluorescence quenching could be categorized into two processes, namely dynamic quenching and static quenching. Dynamic quenching occurs when the quencher collides with biological macromolecules, while static quenching is caused by the formation of complexes. For the dynamic process, the increase in *K*_sv_ along with the increase in temperature is expected, and the opposite is seen for the static quenching process [18]. By examining the interactions between MYR/MYT and PK at temperatures of 290, 300, and 310 K, an apparent linear relationship between *F*_0_/*F* and [*Q*] was achieved, as shown in Figure 3A, indicating that the Stern–Volmer equation quenching constants (*K*_sv_) show a negative correlation with temperature (T). Consequently, we know that the combination of PK and MYR/MYT could form PK-MYR and PK-MYT complexes, leading to the static quenching mechanism. 

Normally, the maximum collisional quenching constant *K*_q_ is about 2 × 10^10^ L·mol^−1^·s^−1^ for small molecule quenchers and biomolecules [19]. If a measured *K*_q_ is much larger than 2 × 10^10^ L·mol^−1^·s^−1^, it indicates that the quenching induced by the quencher is static quenching. Table 1 gives the *K*_q_ values for MYR and MYT at different temperatures, indicating that the quenching processes are both static. Meanwhile, the static quenching constants of PK-MYR exhibit a more remarkable change with increasing temperature, suggesting that the PK-MYR complex is less stable and more sensitive to higher temperature compared to the PK-MYT complex.

**Figure 4:**
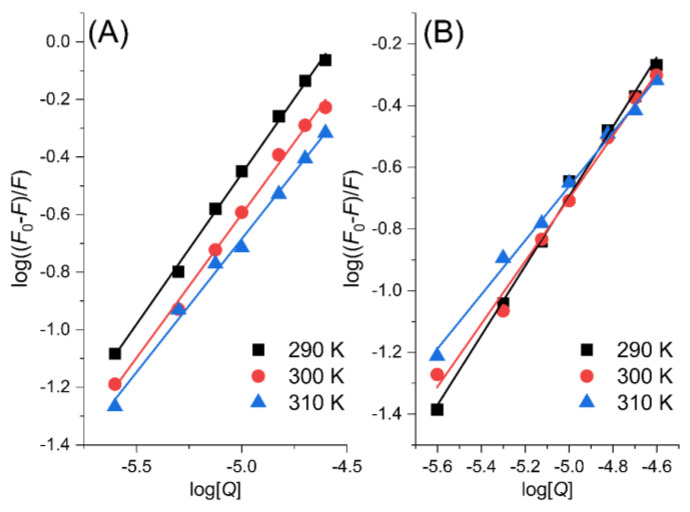
Double−logarithmic plots of PK−MYR (**A**) and PK−MYT (**B**).

The binding constants and the number of binding sites can be calculated by using a double logarithmic equation [20,21] (Equation (3)).

(3) $$\log\left[\frac{\left(F_{0}-F\right)}{F}\right]=\log K_{A}+n_{A}\log\left[Q\right]$$

where *K_A_* represents the binding constant between the biomolecule and the quencher, and *n*_A_ implies the number of binding sites between the quencher and the fluorophor. The binding constants of PK in Figure 4A,B decrease with increasing temperature, suggesting that the stability of PK-MYR and PK-MYT complexes decreases at high temperatures and increasing temperature is not favorable for complex stability. Applying Equation (3) to the data in Figure 4, the *K_A_* and *n*_A_ values could be calculated and are listed in Table 1. It could be proposed that the binding constant of PK-MYT is larger than that of PK-MYR at the temperature of 290 K, and these two complexes share a similar binding site number n around one.

Based on the binding constants *K_A_* at different temperatures, namely 290, 300, and 310 K, respectively, listed in Table 1, the interaction forces between PK and MYR/MYT by the Van’t Hoff equation [22,23], Equations (4) and (5), could be elucidated.

(4) $$\ln K_{A}=-\frac{\Delta H}{RΤ}+\frac{\Delta S}{R}$$

(5) $$\Delta G=-RTInK_{A}=\Delta H-T\Delta S$$

where *R* is the molar gas constant with a general value of 8.314 J·mol^−1^·K^−1^. ∆*H* and ∆*S* are the enthalpy and entropy changes during the fluorescence quenching, respectively. The ∆*G* in Equation (5) is the Gibbs free energy change in the reaction. In general, the interaction between quenchers and fluorophors can be explained by four forces, including hydrogen bonding, van der Waals forces, electrostatic forces, and hydrophobic interactions [24,25,26]. The sign and magnitude of ∆*H* and ∆*S* can be used to distinguish the binding forces and properties of the complexes. When ∆*H* and ∆*S* are positive, hydrophobic interactions dominate; when both ∆*H* and ∆*S* are negative, they can be explained as hydrogen bonding and van der Waals forces; when ∆*H* and ∆*S* are very low negative or positive values, there exist electrostatic interactions.

In Table 2, the negative ∆*G* value illustrates that the processes of PK-MYR and PK-MYT formation are spontaneous. Correspondingly, the negative values of ∆*H* and ∆*S* for both processes indicate that the formation of both complexes is exothermic, where hydrogen bonding and van der Waals forces may dominate the host–guest interactions, and where PK could be considered as the host and the quencher, MYR or MYT, which would be the guest herein. In Table 2, the value of ∆H for PK-MYR is slightly lower than that of PK-MYT, showing that PK-MYT might have higher thermodynamic stability.

### 2.2. Förster Resonance Energy Transfer

The distance and energy transfer between two chromophores, commonly known as the donor and the acceptor, may be computed by the Förster resonance energy transfer (FRET) theory [27]. The UV–Vis absorption spectra of MYR and MYT were chosen to overlap with the fluorescence emission spectra of PK at 290 K [28]. The energy transfer efficiency and binding distance were estimated according to Equation (6).

(6) $$E=1-\frac{F}{F_{0}}=\frac{R_{C}{}^{6}}{R_{C}{}^{6}+r^{6}}$$

where *E* is the energy transfer efficiency between the donor and the acceptor, r is the binding distance between the donor and the acceptor, and *R_C_* is the critical distance when the energy transfer efficiency is 50%, the value of which can be calculated using Equation (7) [29].

(7) $$R_{C}{}^{6}=8.8\times 10^{-25}\left(K^{2}\cdot \Phi \cdot n^{-4}\cdot J\right)$$

where *K*^2^ is the spatial orientation factor associated with the geometry of the dipole donor and acceptor and generally takes the value of 2/3. Φ is the fluorescence quantum yield of the donor. The symbol *n* is the average refractive index of the medium and usually takes the value of 1.336 as the average value of water and organic matter [30]. *J* is the spectral overlap integral between the fluorescence emission spectrum of the donor and the absorption spectrum of the acceptor, and the value is calculated [29] by Equation (8).

(8) $$J=\frac{\sum \left(F_{D}\left(\lambda \right)\cdot \varepsilon \left(\lambda \right)\cdot \lambda^{4}\cdot \Delta \lambda \right)}{\sum \left(F_{D}\left(\lambda \right)\cdot \Delta \lambda \right)}$$

where *F_D_*(*λ*) is the corrected fluorescence intensity of the donor in the wavelength range, *ε*(*λ*) is the molar absorption constant of the acceptor at the wavelength, and ∆*λ* is the span of the wavelength. Following the FRET theory, the main factors affecting the energy transfer efficiency include the fluorescence production of the donor, the overlap of the fluorescence emission spectrum of the donor with the UV–Vis absorption spectrum of the acceptor, and the binding distance between the donor and the acceptor that must be within a specified range of 1 to 10 nm [31].

**Figure 5:**
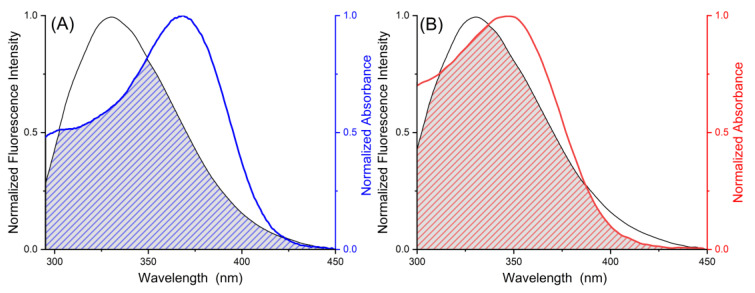
The normalized emission and absorption spectra of the two FRET pairs with schematized spectral overlaps are shown. (**A**) PK (black, donor) and MYR (blue, acceptor), (**B**) PK (black, donor) and MYT (red, acceptor). The concentrations of PK, MYR and MYT are 3.46 μM.

Referring to Figure 5 and Table 3, the binding distances of MYR and MYT are in the range of 0.5 *R*_C_< *r* < 1.5 *R*_C_, which confirms that the binding processes are static quenching processes, which is consistent with the results of fluorescence spectroscopy. The binding distances of MYR and MYT to PK were 5.11 and 3.89 nm, respectively, illustrating the energy transfer from PK to MYR and MYT. Coherently, the binding distance of MYR and PK is larger than that of MYT and PK, indicating that the complex of PK-MYR is not as stable as PK-MYT, which is consistent with the conclusion from the Van’t Hoff equation.

### 2.3. Synchronous Fluorescence Spectroscopy

Synchronous fluorescence spectroscopy studies were performed to observe changes in the molecular environment of Tyr residues and Trp residues in the host structure, which are mainly responsible for fluorescence emission [24,32,33]. Therefore, we used the characteristic information of Tyr and Trp residues, ∆λ = 15 nm and ∆λ = 60 nm, respectively, to probe the changes in the PK-MYR and PK-MYT complexes. 

**Figure 6:**
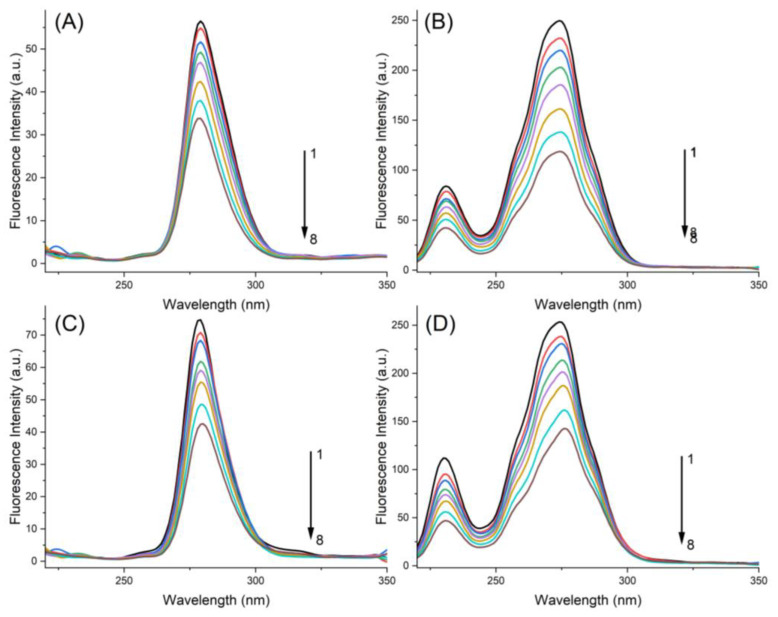
Synchronous fluorescence spectra of different complexes at 300 K. (**A**) PK-MYR (∆λ = 15 nm), (**B**) PK-MYT (∆λ = 15 nm), (**C**) PK-MYR (∆λ = 60 nm), (**D**) PK-MYT (∆λ = 60 nm). From 1 to 8, the concentrations of MYR and MYT are 0.0, 2.5, 5.0, 7.0, 10.0, 15.0, 20.0, and 25.0 μM, respectively.

In Figure 6A, the maximum emission wavelength of PK was retained at 279.0 nm, although the concentration of MYR was increased from 0.0 to 25.0 μM. For the case of the PK-MYT complex shown in Figure 6C, the maximum emission wavelength of PK exhibited a slight red shift from 279.0 to 280.0 nm. Similarly, in Figure 6B,D, the maximum emission wavelength of PK exhibited no shift and a red shift (from 274.0 to 276.0, respectively). 

Normally, the shift of maximum emission wavelength correlates with changes of the hydrophobicity around the chromophores [34]. The red shift indicated that the chromophores moved to a more polar environment, while the blue shift indicated a more hydrophobic environment around the chromophores. These results suggested that the binding phenomenon of MYT to PK induced conformational changes in PK. Meanwhile, the hydrophobic environment of both Tyr and Trp residues gradually decreased according to the addition of MYT.

### 2.4. Competitive Binding Experiments

To determine the binding site of PK, two typical ligands including ibuprofen and cytisine were chosen to perform competitive binding experiments [33,35]. From Figure 6, it can be seen that the fluorescence intensity of PK–ibuprofen and PK–cytisine complexes decreases along with the increasing concentration of MYR and MYT. The site-labeling experiments were still performed using Equations (1) and (2) for correction and data analysis, respectively. As shown in Table 4, *K*_sv_ of PK–ibuprofen and PK–cytisine complexes were only 69.51% and 70.73% of that of free PK as 2.46 × 10^4^ L·mol^−1^, indicating that there exist site competition for both ibuprofen and cytisine in competition with MYR, and suggesting that MYR preferentially binds to the hydrophobic cavity of PK. Similarly, it could be remarked that for MYT, the *K*_sv_ of PK–ibuprofen and PK–cytisine complexes were only 61.52% and 38.14% of that of free PK as 2.06 × 10^4^ L·mol^−1^. These results revealed that both cytisine and ibuprofen compete for the same binding site of PK. It has been well known that neutral compounds such as ibuprofen and cytisine are likely to bind with proteins through hydrophobic interactions and hydrogen binding [36,37]. The competition of MYT was greater than that of MYR, which shows the that binding site for MYT is different to that of MYR. This is consistent with the results obtained from the thermodynamic calculations.

### 2.5. Molecular Docking Study

Molecular docking could be regarded as an appropriate way to propose the plausible sites for protein–ligand interactions. By the help of molecular docking, the conformation and orientation (referred as the “pose”) of ligands into the binding site of a macromolecular target could be analyzed. The pose with the lowest binding energy could be looked as the most likely binding mode of the ligand [38]. Thus, to probe the possible binding modes of MYR and MYT with PK and to better understand the interactions between the host and the guests, molecular docking was used to realize how ligands bind to the host. 

**Figure 7:**
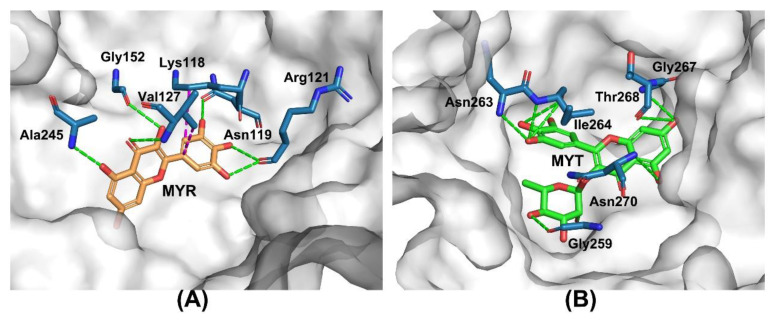
The lowest binding energy poses for PK-MYR (**A**) and PK-MYT (**B**) in the 3D-view cartoon presentation. Green dashes indicate hydrogen binding, and purple dashes indicate hydrophobic interactions between the host and the ligand.

The lowest-binding-energy docking poses are shown in Figure 7, which reveals that MYR fits deeply into PK and is docked into a pocket of PK. Hydrogen binding between MYR and residues of Asn119, Arg121, Val 127, Gly152 and Ala 245 could be responsible for the stabilization of the PK-MYR complex. Hydrophobic interactions might be much weaker and only play an auxiliary role in PK-MYRA complex stability. For the case of MYT, there were four hydrogen bindings between MYT and residues of Gly259, Asn263, Ile264, Gly267, Thr268 and Asn270. There were no hydrophobic interactions found for PK-MYT. It could be seen that MYT has more binding sites and more binding interactions with PK than with MYR. Considering the structural characteristics of PK [39], because MYT has a larger molecular shape and higher hydrophilicity, it might occupy a larger space of docking, have more interactions with polar residues of PK and bind to the host more tightly. This result is consistent with the fluorescence quenching results.

### 2.6. Molecular Dynamics Simulation Study

**Figure 8:**
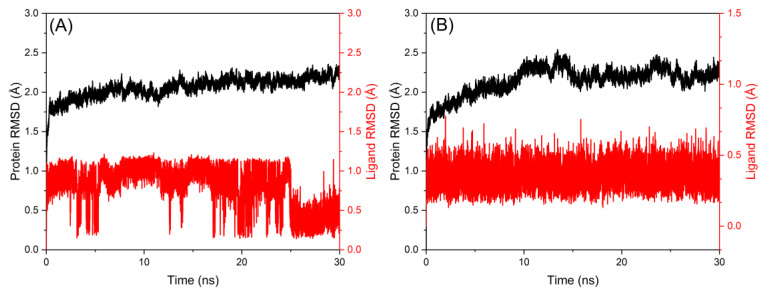
RMSD versus time for the PK-MYR complex (**A**) and PK-MYT (**B**) complex. RMSD for the heavy atoms of PK is shown in black. RMSD for the heavy atoms of MYR (**A**) and MYT (**B**) is shown in red.

Molecular dynamics (MD) is an invaluable technique used to investigate the structural changes of the host in the presence of the ligand as a way to further investigate the stability and flexibility of the host after binding with the ligand [40]. The lowest binding energy poses of PK-MYR and PK-MYT were subjected to 30 ns all-atom MD simulations at 300 K to evaluate the dynamic stability of the complexes. Root mean square deviation (RMSD) of the host and the ligand in PK-MYR and PK-MYT are presented in Figure 8. It is clear that the RMSD of both the host and the ligand in Figure 8A is in rapid increase when the simulation time is in the first 2 ns, illustrating that the confirmations of the host and the ligand are in the process of changing from dynamic instability to stability. During the time from 2 to 17 ns, the RMSD of PK are stable while MYR still fluctuates. The average RMSD of PK and MYR was found at 2.06 and 0.81 Å, respectively, suggesting that MYR was bound to PK, but the stability of MYR was not stable enough after the complex formation. Similarly, as shown in Figure 8B, the RMSD of PK and MYT reached equilibrium at 2 ns, and the RMSD of PK and MYT did not fluctuate drastically from 2 to 30 ns, with averages of 2.15 and 0.36 Å, respectively. The convergence of the RMSD trends demonstrated that the conformational changes of both the host and ligand were small, indicating that the structural stability of the complex is excellent. This result is consistent with the discussions in FRET.

## 3. Materials and Methods

### 3.1. Materials

PK was obtained from Jiuding Chemicals (Shanghai, China). Both MYR and MYT were purchased from Kailai Biological Co., Ltd. (Xi’an, China). Tris(hydroxymethyl)aminomethane (Tris) was purchased from Amresco (Solon, OH, USA). HCl and ethanol were purchased from Sinopharm Chemical Reagent Co., Ltd. (Shanghai, China). Ibuprofen and cytisine were sourced from Macklin Biotechnology (Shanghai, China) and Yuanye Biotechnology (Shanghai, China), respectively. The reagents were of analytical grade or higher, stored at 0–4 °C, and used directly without extra pretreatment. Ultrapure water (18.2 MΩ·cm) from an Elga PureLab Classic system was used for the solution preparation and dilution.

### 3.2. Instruments

A Cary Eclipse fluorescence spectrometer (Varian, Palo Alto, CA, USA) was used to determine specific changes in the fluorescence emission of PK at different temperatures. All measurements were recorded at an excitation wavelength of 278 nm and the emission wavelength of 290–450 nm [41]. The excitation and emission slits were adjusted to 5 nm. In synchronous fluorescence measurements, ∆λ was set to 60 and 15 nm. Typically, a 25 mM Tris-HCl (pH 8.0) solution containing 3.46 μM PK and 0–25 μM MYR or MYT at 290, 300, or 310 K is used for fluorescence spectroscopy measurements.

UV–Vis absorption spectra were recorded by using a UH5300 UV–Vis spectrometer (Hitachi, Japan) at wavelengths ranging from 225 to 450 nm, with a band width of 1 nm and data interval of 0.5 nm [42].

### 3.3. Site-Tagged Competition Experiments with Ibuprofen and Cytisine

For in site labeling competition experiments, an agent such as ibuprofen or cytisine of 3.46 μM was first mixed with PK. Then, different concentrations of MYR and MYT (0–25 μM) were added dropwise to the mixture and incubated at room temperature for 1 h. The excitation wavelengths for PK–ibuprofen and PK–cytisine were 265 and 280 nm, respectively. Fluorescence spectra were analyzed using the Stern–Volmer equation.

### 3.4. Förster Resonance Energy Transfer Studies

Förster resonance energy transfer (FRET) was tracked by overlapping PK emission spectrum (donor) with MYR or MYT absorption spectrum (acceptor). A concentration of 3.46 μM PK was chosen for the fluorescence emission spectroscopy measurements. After that, the UV–Vis absorption spectra were collected by the molar ratio of 1:1 type PK and MYR/MYT mixture, and the reference was secondary deionized water.

### 3.5. Molecular Docking Studies

Three-dimensional structure of PK was obtained from the RCSB Protein Data Bank [43] with the entry 2ID8 (https://doi.org/10.2210/pdb2ID8/pdb) (accessed on 15 February 2023). Structural data of MYR (PubChem CID: 5281672) or MYT (PubChem CID: 5281673) were obtained from PubChem (https://pubchem.ncbi.nlm.nih.gov) (accessed on 15 February 2023) and optimized by the MMFF94 force field [44]. Molecular docking studies were performed by using AutoDock 4.2 (Scripps Research Institute, San Diego, CA, USA) package [45]. Rotatable bonds and non-polar hydrogen atoms of the ligands, namely MYR and MYT, were kept according to the default setting of Autodock. The size of the cubic grid for docking was set as 126 × 126 × 126 Å^3^, and the spacing of the grid was 0.375 Å. To find the optimal binding site for PK and the ligand, the Lamarckian Genetic Algorithm (LGA) with parameters set to 300 GA operations at a maximum number of energy evaluations of 2.5 × 10^6^, population size set to 150, and the rest of parameters were used as default values. Molecular docking results were analyzed using Protein–Ligand Interaction Profiler (PLIP) [46].

### 3.6. Molecular Dynamics Studies

In this work, molecular dynamics of highest-scored molecular docking results for the PK-MYR and PK-MYT complexes were performed using the free package for molecular dynamics Desmond 2018.04 (DE Shaw Research, NY, USA) [47]. The energy-optimal molecular docking results were introduced into the center of a 10 × 10 × 10 Å cubic box, which was subsequently filled with TIP3P water molecules. To neutralize the solvent system, 5 Cl^−^ as the counter ions were automatically added by Desmond. The system energy minimization and system relaxation processes were achieved using the Desmond default parameter setting procedure. The NPT ensemble was used, with a simulated temperature of 300 K and a pressure of 1.01325 bar, the temperature was controlled by the Nose-Hoover coupling method with a relaxation time of 1 ps, and the pressure was controlled by the Martyna–Tobias–Klein method with a typical relaxation time of 2 ps. The isotropic pressure coupling was used, and the integration step of the simulation was set to 2 fs. After 1 ns equilibrium, molecular dynamics simulations of the system were performed for 30 ns.

## 4. Conclusions

In this work, PK was used as a fluorescent agent, and both MYR and MYT were selected as quenchers for the fluorescence spectroscopy and molecular simulation studies. By fluorescence spectroscopic studies, it was clear that the formations of PK-MYR and PK-MYT complexes are both processes of static quenching. The binding sites and binding constants of the complexes could be calculated by using double logarithmic equations. Compared with MYR, MYT has more binding sites and is closer to the Trp residue in the complex. Moreover, temperature affects the stability of the complexes of PK-MYR and PK-MYT. High temperature could decrease the complex stability, especially for PK-MYR. The thermodynamic calculations revealed that *∆H* and *∆S* for both processes were both negative, indicating that the complex formation processes are all exothermic. Hydrogen bonding and van der Waals forces are the main driving forces for complex formations. Negative *∆G* values demonstrate that these complex formations are spontaneous processes. FRET experiments showed that MYT might bind closer to PK and has a higher energy transfer efficiency than MYR. Simultaneous fluorescence spectroscopy proves the conformational change of PK in the local environment of amino acid residues due to a change in polarity around Tyr residues, leading to a decrease in hydrophobicity. Molecular docking results help to understand the binding mode and host–ligand interactions between PK and MYR/MYT. Molecular dynamics results revealed the stability of the host and the ligands after a 30 ns simulation. It is clear that MYT has higher stability and low flexibility when bound to PK. These results are of practical significance and are reference values for the pharmacological research and development of MYR and MYT.

## Figures and Tables

**Table 1:** Calculated Stern–Volmer constant (*K*_sv_), fluorescence quenching rate constant (*K*_q_), binding constant (*K*_A_), and number of binding sites (*n*_A_) for the interactions of PK with MYR and MYT at different temperatures (T).

Ligand	*T*/K	*K*_sv_/10^4^ L·mol^−1^	*K*_q_/10^12^ L·mol^−1^·s^−1^	*K*_A_/10^4^ L·mol^−1^	*n*_A_
MYR	290	3.57	3.57	5.90	1.0
	300	2.46	2.46	2.47	0.9
	310	1.90	1.90	0.88	0.9
MYT	290	2.20	2.20	8.78	1.1
	300	2.06	2.06	2.43	1.0
	310	1.88	1.88	0.53	0.9

**Table 2:** Calculated thermodynamic quantities including enthalpy, entropy and Gibbs free energy changes shown with Δ*H*, Δ*S* and Δ*G*, respectively, for the PK-MYR and PK-MYT complexes.

Ligand	T/K	∆*H*/kJ·mol^−1^	∆*S*/J·mol^−1^·K^−1^	∆*G*/kJ·mol^−1^
MYR	290	−98.01	−3.51	−27.62
	300			−25.23
	310			−22.77
MYT	290	−105.01	−3.86	−27.44
	300			−25.18
	310			−22.09

**Table 3:** Calculated spectral overlap integral (*J*), critical distance (*R*_C_), energy transfer efficiency (*E*) and binding distance (*r*) for PK and different ligands.

Ligand	*J*/cm^3^∙L∙mol^−1^	*R*_C_/nm	*E*	*r*/nm
MYR	9.29 × 10^−^^14^	3.61	0.105	5.11
MYT	6.73 × 10^−^^14^	3.42	0.128	3.89

**Table 4:** The Stern–Volmer equation quenching constant (*K*_SV_) for the site-labeling competition experiments of PK-MYR and PK-MYT complexes.

Ligand	Site Marker	*K*_SV_/10^4^ L·mol^−1^	*R* ^a^
MYR	blank	2.46	0.992
	ibuprofen	1.71	0.998
	cytisine	1.74	0.995
MYT	blank	2.06	0.997
	ibuprofen	1.27	0.998
	cytisine	0.78	0.994

^a^ *R* is the correlation coefficient.

## Data Availability

Not applicable.
